# Correlation analysis between subtest scores of CERAD-K and a newly developed tablet computer-based digital cognitive test (Inbrain CST)

**DOI:** 10.3389/fnagi.2023.1178324

**Published:** 2023-06-29

**Authors:** Seunghee Na, Sang Won Seo, Young Ju Kim, Heejin Yoo, Eek-Sung Lee

**Affiliations:** ^1^Department of Neurology, Incheon St. Mary’s Hospital, The Catholic University of Korea, Seoul, Republic of Korea; ^2^Department of Neurology, Samsung Medical Center, Sungkyunkwan University School of Medicine, Seoul, Republic of Korea; ^3^Department of Neurology, Soonchunhyang University Bucheon Hospital, Bucheon, Republic of Korea

**Keywords:** neuropsychological test, computerized cognitive test, CERAD-K, cognitive impairment, mild cognitive impairment, correlation study

## Abstract

**Introduction:**

The prevalence of Alzheimer’s disease (AD) and other dementias is increasing; therefore, identifying individuals at risk for dementia is crucial. Traditional neuropsychological assessments are expensive and time-consuming; however, computerized cognitive testing is becoming popular in clinical and research settings, particularly during the COVID-19 pandemic. This study aimed to investigate the correlation between the computerized cognitive test, Inbrain cognitive screening test (CST), and the traditional neuropsychological battery, the consortium to establish a registry for Alzheimer’s disease assessment packet (CERAD-K).

**Methods:**

We enrolled 166 participants from five districts in Republic of Korea, including cognitively unimpaired individuals and those with mild cognitive impairment (MCI) diagnosed by experienced neurologists. We used the Inbrain CST and CERAD-K to evaluate the cognitive function of the participants, and the scores of each subtest of the Inbrain CST and CERAD-K were compared.

**Results:**

A significant correlation was found between the Inbrain CST and CERAD-K subtests. Furthermore, multivariate analysis revealed a significant correlation between the Inbrain CST and the CERAD-K test pairs after adjusting for age, educational level, and sex.

**Discussion:**

In conclusion, this study demonstrates that the Inbrain CST is a reliable tool for detecting cognitive impairment in cognitively unimpaired individuals and patients with MCI, because it has a high correlation and agreement with CERAD-K. Therefore, the Inbrain CST can be a useful, time-efficient, and cost-effective computer-based cognitive test for individuals at risk for cognitive impairment.

## 1. Introduction

The number of patients living with Alzheimer’s disease (AD) and other types of dementia continues to grow with the increasing population of individuals aged ≥ 65 years. Although deaths from cardiovascular diseases have decreased since 2000, deaths from AD have increased by approximately 1.5 times (The Alzheimer’s Association, 2022). Anti-amyloid monoclonal antibodies are being actively investigated as a potential treatment of AD (Dhillon, 2021; van Dyck et al., 2023); therefore, the importance of detecting patients at risk of dementia cannot be overemphasized.

Identifying objective cognitive impairment is an essential component of various neurocognitive disorders. However, conducting a traditional neuropsychological assessment with pencil and paper is costly, time-consuming, and requires specialized skills for both administration and interpretation (Echemendia et al., 2013). Therefore, the call for computerized neuropsychological assessments has persisted for both clinical and research purposes (Bauer et al., 2012). Moreover, since the COVID-19 era, there has been a growing need for assessment methods that do not require close contact with patients experiencing cognitive impairment. Computerized cognitive tests can prove to be effective in this regard, although some level of monitoring during the testing process may be necessary. Neuropsychological assessment instruments facilitated by computing technologies confer multiple benefits. These encompass the ability to rapidly administer tests to a substantial number of subjects, potentially curtail evaluation duration via adaptive testing methodologies, expand service accessibility to patients situated in regions or conditions where professional neuropsychological resources are limited, and the proficiency to incorporate and mechanize interpretive algorithms (Bauer et al., 2012). As such, computerized neuropsychological assessments can considerably reduce the need for human, material, and time resources typically required by traditional neuropsychological tests. Consequently, these computerized methods can be less costly and time-consuming, and demand fewer specialized skills compared to pencil and paper assessments.

Several validation and correlation studies have been conducted to compare computerized cognitive testing and traditional neuropsychological assessments in the general population (Kataja et al., 2017; Cole et al., 2018; Busch et al., 2019), patients with infection or traumatic brain injury (Cole et al., 2018; De Francesco et al., 2021), and patients with various neurodegenerative disorders (Hammers et al., 2012; Busch et al., 2019). However, there is a lack of correlation study between computerized cognitive testing and the pencil-and-paper neuropsychological battery in terms of all cognitive domains among patients with cognitive impairments. A computer-based cognitive screening test, the Inbrain cognitive screening test (CST), was previously validated across the following populations: cognitively normal, subjective cognitive decline, mild cognitive impairment (MCI), and dementia (Chin et al., 2020). The Inbrain CST was well correlated with the scores of the Seoul Neuropsychological Screening Battery (SNSB) (Ryu and Yang, 2023), which is a widely used traditional pencil-and-paper test in Republic of Korea (Chin et al., 2020).

The consortium to establish a registry for Alzheimer’s disease assessment packet (CERAD) is one of the traditional neuropsychological batteries. The CERAD was designed to detect the early cognitive alterations associated with AD and translated into various languages including Korean (Lee et al., 2002). The Korean version of CERAD (CERAD-K) has been adopted as a standard neuropsychological battery along with SNSB and has been actively utilized in both clinical and research settings. The comparison of computerized cognitive assessment to that standard neuropsychological test, CERAD-K, may increase the reliability and applicability, and also be useful to expand its use of the research field. Thus, we investigated the correlation of each test in the Inbrain CST with the CERAD-K.

## 2. Materials and methods

### 2.1. Participants

Participants were enrolled as part of a prospective community-based cohort study for MCI (PREcision medicine platform for mild cognitive impairment on multi-omics, imaging, and evidence-based R&BD: PREMIER). Cognitively unimpaired individuals and patients with MCI were enrolled in the PREMIER cohort study. A total of 166 individuals who visited the Center for Dementia of the Bucheon, Dongtan, Gangnam, Gijang, and Haeundae districts in Republic of Korea were recruited. The Center of Dementia is a public health center-affiliated organization for integrated dementia management that combines education on dementia prevention, cognitive screening, cognitive rehabilitation programs, and support for patients with dementia and their caregivers. All participants were evaluated by experienced neurologists and underwent comprehensive neuropsychological tests, including the CERAD-K (Lee et al., 2002) and the Inbrain CST.

Neurologists diagnosed the patients with MCI when they fulfilled the following inclusion criteria based on Petersen’s definition (Petersen, 2004): (1) memory complaints observed by an informant; (2) objective impairment in memory function relative to the individual’s age, sex, and education; (3) preserved general cognitive function; and (4) largely intact activities of daily living. We determined objective impairment in memory function when the participants scored 1.5 standard deviations below in at least one of the four episodic memory test scores in the CERAD-K, including word list memory, word list recall, word list recognition, and constructional praxis recall tests. Written informed consent was obtained from all participants, and this study was approved by the Institutional Review Board of Soonchunhyang University Buchen Hospital (IRB approval No. 2017-04-058).

### 2.2. Inbrain CST, a computerized cognitive test

The Inbrain CST was run on a 12-inch tablet PC using Microsoft Windows 10. This program was composed of seven neuropsychological tests assessing the five cognitive functions, including the forward and backward visual span test (VST) for attention domain, the Difficult Naming Test (DNT) and semantic (fruits)/phonemic (Korean alphabet digeut) word fluency test for language domain, the block design test for visuospatial function, the time orientation tests and word place association test (WPAT) for memory domain, and the Korean trail-making test-Elderly version (K-TMT-E) part A and B for executive domain. Age-, sex-, and education-specific norms were provided for each cognitive domain, as in a previous study of 480 elderly, cognitively normal, and community-dwelling individuals (Chin et al., 2020). Before each task, participants received brief verbal and written instructions, and the examiner monitored the entire session of the test. Furthermore, all participants completed the test without any noticeable interference from the examiner.

### 2.3. CERAD-K

The CERAD-K is a Korean-translated clinical and neuropsychological assessment battery (Lee et al., 2002). It consists of nine subtests for assessing five cognitive domains: the Korean version of the mini-mental state examination (K-MMSE) for general cognitive function; Verbal Fluency and Boston Naming Test for language domain; word list memory, word list recall, word list recognition, and constructional praxis recall for memory domain; constructional praxis for visuospatial domain; trail-making test A for attention; and trail-making test B for executive function. Normative data were stratified by age, sex, and educational level. The norm was offered scores of the median and fifth percentile for each task and was determined as “abnormal” when the score was below the fifth percentile.

### 2.4. Statistical analysis

Before the comparison, we paired subtests of the Inbrain CST with those of CERAD-K as follows (Table 3): DNT and the Boston Naming Test, the semantic (fruits) word fluency test and controlled oral word association test, the block design test and construction praxis test, WPAT-immediate recall and word list memory test, WPAT-delayed recall and word list recall, WPAT-word recognition and word list recognition test, and WPAT-place recognition and constructional praxis recall tests. The trail-making test part A and part B in the CERAD-K were performed in 152 and 76 participants, respectively. Spearman’s correlation analysis was performed to examine the correlation between the Inbrain CST and CERAD-K subtests. Statistical analysis was performed using SAS version 9.4 (SAS Institute Inc., Cary, NC, USA) and R 4.2.1 (Vienna, Austria),^1^ and differences were considered statistically significant at *p* < 0.05. Furthermore, the degree of correlation was regarded as very high if the coefficient value was 0.9–1, high if the value was 0.7–0.9, moderate if the value was 0.5–0.7, low if the value was 0.3–0.5, or negligible if the value was < 0.3 (Mukaka, 2012).

## 3. Results

The baseline characteristics of the study participants are presented in Table 1. The 166 participants included 73 cognitively unimpaired individuals and 93 patients with MCI. The participants were aged 72.6 ± 8.6 years on average and 113 (68.1%) individuals were women. Additionally, the average education level was 7.9 ± 4.1 years. In this population, the K-MMSE was 23.94 ± 4.54, and the global score of clinical dementia rating (CDR) and CDR-sum of boxes were 0.38 ± 0.22 and 1.12 ± 1.02, respectively. Participants with cognitively unimpaired status exhibited a K-MMSE score of 26.7 ± 3.3, a global CDR score of 0.25 ± 0.25, and a CDR sum of boxes score of 0.59 ± 0.76. The patients with MCI demonstrated a K-MMSE score of 21.78 ± 4.18, a global CDR score of 0.48 ± 0.13, and a CDR sum of boxes score of 1.53 ± 1.01.

**TABLE 1:** Baseline demographic and clinical characteristics of the participants.

Characteristics	Participants		
Age (years)	72.60 ± 8.63		
Education (years)	7.86 ± 4.05		
Sex (female)	113 (68.1%)		
Hypertension	82 (49.4%)		
Diabetes	38 (22.9%)		
Dyslipidemia	66 (39.8%)		
Coronary artery disease	14 (8.4%)		
**Participants group**	**Total**	**Cognitively unimpaired**	**MCI**
	166	73 (44.0%)	93 (56.0%)
K-MMSE	23.94 ± 4.54	26.68 ± 3.29	21.78 ± 4.18
CDR	0.38 ± 0.22	0.25 ± 0.25	0.48 ± 0.13
CDR-sum of boxes	1.12 ± 1.02	0.59 ± 0.76	1.53 ± 1.01

Data are expressed as mean ± standard deviation or *n* (%). K-MMSE, Korean version of the mini-mental state examination; CDR, clinical dementia rating; MCI, mild cognitive impairment.

The results of the Inbrain CST tests and correlation analysis of the z-scores of the subtests between the Inbrain CST and CERAD-K are shown in Tables 2, 3. Regarding Inbrain CST, the z-scores of the semantic word fluency test (*r* = −0.352, *p* < 0.0001), Difficult Naming Test (*r* = −0.309, *p* < 0.0001), block design test (*r* = −0.194, *p* = 0.0124), and immediate recall (*r* = −0.345, *p* < 0.0001), delayed recall (*r* = −0.368, *p* < 0.0001), and word recognition tests (*r* = −0.291, *p* = 0.0001) of WPAT revealed a significant negative correlation with age. Additionally, all subtests of the Inbrain CST, except for the block design test, were significantly correlated with educational level. However, sex had no statistical effect on any of the Inbrain CST subtests.

**TABLE 2:** The correlation between subtests of the Inbrain CST and demographic variables.

Subtests of Inbrain CST	Z-scores	Variables	*r*	*P*-value
Semantic word fluency test	−0.44 [−1.11, 0.76]	Age	-0.352	< 0.0001
		Education level	0.324	< 0.0001
		Sex		0.168
Difficult Naming Test	−0.04 [−0.99, 0.78]	Age	-0.309	< 0.0001
		Education level	0.326	< 0.0001
		Sex		0.3485
Immediate recall of WPAT	−0.54 [−1.61, 0.65]	Age	-0.345	< 0.0001
		Education level	0.374	< 0.0001
		Sex		0.4413
Block design test	−0.25 [−0.85, 0.41]	Age	-0.194	0.0124
		Education level	0.127	0.1024
		Sex		0.0513
Delayed recall of WPAT	−0.69 [−2.14, 0.46]	Age	-0.368	< 0.0001
		Education level	0.368	< 0.0001
		Sex		0.2187
Word recognition of WPAT	−0.65 [−2.35, 0.37]	Age	-0.291	0.0001
		Education level	0.310	< 0.0001
		Sex		0.8788

Z-scores are presented as median [interquartile range]. Correlation analysis between each subtest, age, and education level were conducted using Spearman’s correlation analysis. Statistical analysis of the relationship between sex and each subtest was performed using a *t*-test or Wilcoxon rank-sum test. CST, cognitive screening test; WPAT, word place association text.

**TABLE 3:** The correlation between subtests of the Inbrain CST and CERAD-K.

Subtests		Univariate analysis		Multivariate analysis	
**Inbrain CST**	**CERAD-K**	***r***	***P*-value**	***r***	***P*-value**
Semantic word fluency test	Verbal Fluency	0.517	< 0.0001	0.425	< 0.0001
Difficult Naming Test	Boston Naming Test	0.655	< 0.0001	0.603	< 0.0001
Immediate recall of WPAT	Word list memory	0.709	< 0.0001	0.632	< 0.0001
Block design test	Constructional praxis	0.350	< 0.0001	0.345	< 0.0001
Delayed recall of WPAT	Word list recall	0.724	< 0.0001	0.650	< 0.0001
Word recognition of WPAT	Word list recognition	0.631	< 0.0001	0.579	< 0.0001
Trail-making test part A	Trail-making test part A	0.344	< 0.0001	0.228	0.005
Trail-making test part B	Trail-making test part B	0.413	< 0.0001	0.364	0.002

Multivariate analysis was performed after adjusting for age, education level, and sex. K-MMSE, Korean version of the mini-mental state examination; WPAT, word place association test; CST, cognitive screening test.

**FIGURE 1:**
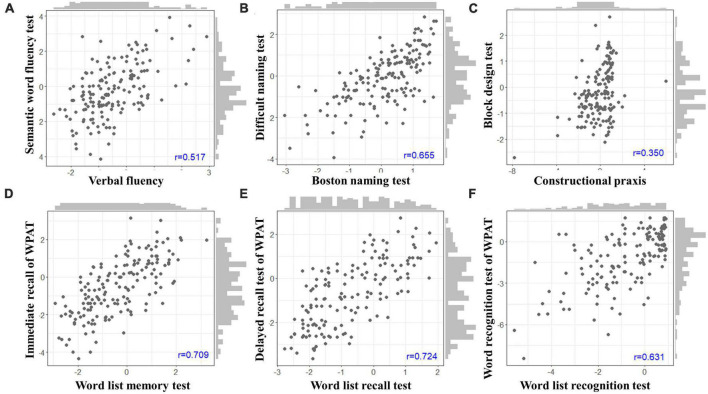
Scatterplots depicting the correlations between the subtest pairs of the Inbrain CST (*y*-axis) and CERAD-K (*x*-axis). **(A)** The semantic word fluency test and Verbal Fluency. **(B)** The Difficult Naming Test and the Boston Naming Test. **(C)** The block design test and the constructional praxis test. **(D)** The immediate recall of WPAT and word list memory test. **(E)** The delayed recall test of WPAT and word list recall test. **(F)** The word recognition test of WPAT and the word list recognition test. WPAT, word place association test; CST, cognitive screening test; CERAD-K, consortium to establish a registry for Alzheimer’s disease assessment packet.

Univariate analysis showed significant correlations between all subtest pairs in the Inbrain CST and CERAD-K. The results are presented in Table 3 and Figure 1. The immediate recall (*r* = 0.709, *p* < 0.0001) and delayed recall (*r* = 0.724, *p* < 0.0001) scores of the WPAT were highly correlated with the scores of the word list memory and word list recall tests of the CERAD-K. The pairs of semantic word fluency test and Verbal Fluency (*r* = 0.517, *p* < 0.0001), Difficult Naming Test and Boston Naming Test (*r* = 0.655, *p* < 0.0001), and word recognition test of WPAT and word list recognition test (*r* = 0.631, *p* < 0.0001) showed moderate correlations. The block design test (*r* = 0.350, *p* < 0.0001) showed a weak correlation with the constructional praxis test. Trail-making test part A (*r* = 0.344, *p* < 0.0001) and part B (*r* = 0.413, *p* < 0.0001) in Inbrain CST also correlated with those of CERAD-K.

Multivariate analysis, after adjusting for age, education level, and sex, revealed a significant correlation between the Inbrain CST and CERAD-K test pairs. In fact, the Difficult Naming Test (*r* = 0.603, *p* < 0.0001), immediate recall (*r* = 0.632, *p* < 0.0001), delayed recall (*r* = 0.650, *p* < 0.0001), and word recognition tests (*r* = 0.579, *p* < 0.0001) of the WPAT revealed moderate correlations with each paired subtest of the CERAD-K. Additionally, the semantic word fluency (*r* = 0.425, *p* < 0.0001), block design tests of the Inbrain CST (*r* = 0.345, *p* < 0.0001), and the trail-making test part B (*r* = 0.362, *p* = 0.0016) showed a low degree of correlation with the corresponding measures of the CERAD-K. The correlation between Inbrain CST and the CERAD-K for trail-making test part A remained significant (*r* = 0.228, *p* = 0.0052).

## 4. Discussion

Our study showed that subtests of the Inbrain CST have good correlations with each counterpart task of the traditional neuropsychological test, CERAD-K, across patients with MCI and cognitively unimpaired individuals. These findings are consistent with those of a previous validation study of the CST, which demonstrated a relationship between the Inbrain CST and another pencil-and-paper neuropsychological battery, the SNSB (Chin et al., 2020).

This study revealed fair to high correlations between the Inbrain CST and CERAD-K subtests, which were matched in terms of the cognitive domains for assessing language, memory, executive, and visuospatial functions. Several correlation and validation studies of the computer-based cognitive tests have been performed. However, several studies have addressed specific cognitive tasks in only one or two cognitive domains (Busch et al., 2019; Sacco et al., 2019), whereas we investigated the correlations in the tasks of all cognitive domains. A previous report has indicated that a computerized brief screening battery that can be used for patients with dementia showed modest correlations with conventional test scores. However, it can only be used for screening purposes because it might be too simple to offer cognitive domain scores or differentiate patients with MCI from cognitively unimpaired or dementia individuals (Hammers et al., 2012). Previous studies have been conducted on the general population or specific patient groups other than those with neurodegenerative disorders (Kataja et al., 2017; Cole et al., 2018; De Francesco et al., 2021); therefore, the application of the tests to patients with cognitive impairment can be limited because the use of the transferred tests on the computer has not been validated for those individuals.

All subtests analyzed in the Inbrain CST exhibited significant correlations with the corresponding measures of the CERAD-K, after adjusting for age, sex, and education. However, several items, such as the semantic word fluency test, the block design test, and trail-making test parts A and B, showed weak correlations (*r* < 0.5). This may be attributed to the varying difficulty levels of each task. For instance, when comparing the block design test and the construction praxis test, the scores of the block design test displayed a wider distribution than those of the construction praxis test (Figure 1). The semantic word fluency tests in the two test batteries are fundamentally identical, except the subject was sitting in front of either the examiner or a device. Thus, the difference between the two tests might be the influence of the test environment, a possible psychological factor induced by the unfamiliarity of the device.

We have found that the Inbrain CST demonstrates a good correlation with the CERAD-K in all aspects of episodic memory evaluation, including the immediate, delayed, and recognition tests. Notably, identifying the pattern of episodic memory impairment helps understand the etiology of cognitive impairment. In general, patients with AD show prominent deficits in both free recall and recognition components of episodic memory tests (Delis et al., 1991; Weintraub et al., 2012). These memory impairment patterns usually precede the onset of functional decline in AD (Chen et al., 2001; Lange et al., 2002). Patients with dementia with Lewy bodies (DLB) tend to perform better on recognition tasks than those with AD, although patients with both DLB and AD can show similar impairments on delayed free recall tests (Hamilton et al., 2004). Compared to patients with AD, those with subcortical vascular dementia often show relatively better recognition performance in free recall-based tests and less severe impairment in the delayed free recall task (Graham et al., 2004; Ramirez-Gomez et al., 2017). Thus, the Inbrain CST can be useful in identifying objective cognitive impairments as well as specific characteristics of cognitive decline.

This study has several limitations. First, we conducted the correlation study across cognitively unimpaired individuals and those with mild cognitive impairment (MCI) together, rather than separately. Considering that cognitive function tests should be administered specifically to patients with cognitive impairment, further studies are needed to include patients with dementia and conduct the analysis with stratification ranging from normal cognitive function to dementia status. Second, the results of the trail-making test in about half of the participants were missing, thus the interpretation of the correlation results of the trail-making test should be considered this aspect. Nevertheless, our study has a strength in a direct comparison between each task in Inbrain CST and the CERAD-K. Beyond the discrimination ability of subjects with cognitive impairment from cognitively unimpaired (Chin et al., 2020), this study supported that computerized cognitive testing may provide reliable and comparable results across the major cognitive domains.

In conclusion, the Inbrain CST, a tablet PC-based digital cognitive test, showed a good correlation with CERAD-K, a representative comprehensive pencil-and-paper version of the neuropsychological test, in all cognitive domains in both cognitively unimpaired participants and those with mild cognitive impairment.

## Data availability statement

The raw data supporting the conclusions of this article will be made available by the authors, without undue reservation.

## Ethics statement

The studies involving human participants were reviewed and approved by the Institutional Review Board of Soonchunhyang University Buchen Hospital (IRB approval No. 2017-04-058). The patients/participants provided their written informed consent to participate in this study.

## Author contributions

E-SL designed the study, conducted research, and had primary responsibility for final content. SS and YK interpreted the data and performed critical review of the manuscript. HY performed statistical analysis and critical review of the manuscript. SN interpreted the data and drafted the manuscript. All authors have read and approved the final manuscript.

## Footnotes

1
http://www.R-project.org
